# Lifetime healthcare expenditures across socioeconomic groups in Sweden

**DOI:** 10.1093/eurpub/ckad140

**Published:** 2023-08-30

**Authors:** Stephanie Fledsberg, Mikael Svensson, Naimi Johansson

**Affiliations:** Health Economics and Policy, School of Public Health and Community Medicine, Institute of Medicine, University of Gothenburg, Gothenburg, Sweden; Gothia Forum for Clinical Trials, Region Västra Götaland, Sahlgrenska University Hospital, Gothenburg, Sweden; Health Economics and Policy, School of Public Health and Community Medicine, Institute of Medicine, University of Gothenburg, Gothenburg, Sweden; Department of Pharmaceutical Outcomes & Policy, College of Pharmacy, University of Florida, Gainesville, FL, USA; Health Economics and Policy, School of Public Health and Community Medicine, Institute of Medicine, University of Gothenburg, Gothenburg, Sweden; Faculty of Medicine and Health, University Health Care Research Center, Örebro University, Örebro, Sweden

## Abstract

**Background:**

Individuals of lower socioeconomic status generally have higher healthcare expenditures than individuals of higher socioeconomic status. However, little is known about how expenditures are distributed across socioeconomic groups over a lifetime, once accounting for differences in life expectancy. This study describes how lifetime healthcare expenditures are distributed across age, sex and socioeconomic groups in Sweden while adjusting for differences in life expectancy.

**Methods:**

Healthcare utilization from 2016 were linked to demographic and socioeconomic data for a random sample of individuals aged 20 and above in the four largest Swedish regions (*n* = 440 659). Mortality data were used to estimate income- and sex-specific survival rates. Expected lifetime healthcare expenditures were estimated by combining survival rates with mean healthcare expenditures over age, by sex, and income quintile.

**Results:**

We find that expected lifetime healthcare expenditures are highest among the first (lowest) income quintile despite their evident lower life expectancy. Expected lifetime expenditures were 17.9% (16.8%) higher in the first income quintile compared to the fifth (highest) quintile for women (men). Individuals in the first income quintile had higher expected lifetime expenditures for all care categories except for primary care.

**Conclusion:**

We conclude that despite a lower life expectancy, the quintile of the lowest socioeconomic status still had higher lifetime healthcare expenditures.

## Introduction

Financial resources spent on healthcare have increased in most high-income countries over the recent decades, rising concern over long-term financial sustainability.^1–3^ Population growth and a changing population age structure have led to an increase in welfare needs, including healthcare services.^4^^,^^5^ Considering containment of health expenditures, the interest in understanding how healthcare utilization and costs are distributed across socioeconomic groups has increased.^2^^,^^3^^,^^6^^,^^7^ Knowledge of the distribution of healthcare expenditures from a lifetime perspective is an essential aspect of efficient resource allocation. Further, improved benchmarks of age and sex-standardized healthcare expenditures estimates can be used to inform economic models seeking to assess future needs or the efficiency of new interventions.^8–11^

A large literature has documented that health differs by socioeconomic status^10–12^—lower socioeconomic groups generally have poorer health and shorter average life expectancy.^12–14^ Over the past 30 years, these differences have tended to increase rather than decrease between socioeconomic groups.^15^^,^^16^ Besides differences in health and mortality risks, groups of lower socioeconomic status, in general, incur increased healthcare expenditures compared with higher socioeconomic groups.^6^^,^^17–19^

While prior studies have estimated healthcare expenditures related to specific illnesses or populations using a lifetime perspective,^1^^,^^8–10^ only a few studies have explored how healthcare expenditures are distributed across socioeconomic groups while considering differences in life expectancy.^2^^,^^7^ A recent study of socioeconomic differences in healthcare expenditures in the Netherlands found that individuals with lower income and education had higher healthcare expenditures for most types of care than individuals of higher socioeconomic status.^19^ However, a Danish study suggested that differences in lifetime healthcare expenditures between socioeconomic groups are lower than expected once accounting for socioeconomic differences in mortality.^2^

In the Swedish setting, a previous study has acknowledged age and sex differences in healthcare consumption,^20^ while little attention has been paid to the extent of socioeconomic differences in healthcare expenditures using a lifetime perspective. Using unique individual-level health data provided by national healthcare registers, this paper aims to describe how lifetime healthcare expenditures of inpatient, specialized outpatient, primary care and prescription drugs are distributed across age, sex and socioeconomic groups in Sweden. To make a relevant comparison of expected lifetime expenditures across socioeconomic groups, we account for differences in life expectancy by sex and income.

## Methods

### Population and data

This cross-sectional study uses pseudonymized individual-level register data based on a random sample of 1 million individuals living in Sweden, equal to approximately 10% of the Swedish population, followed from 2007 to 2016. Data were collected from the National Patient Register, the Prescribed Drug Register, Statistics Sweden’s The longitudinal integrated database for health insurance and labour market studies (LISA) including population statistics and four regional healthcare databases. This paper uses a subset of the original sample, restricting the sample to the year 2016 and the 440 659 adult individuals (age 20 years and above) living in the four largest regions in Sweden: Stockholm, Västra Götaland, Skåne and Östergötland. The four regions account for 57% of the Swedish population and do not stand out in terms of demographic characteristics (Supplementary Appendix table A1). We use these regions since data on primary healthcare utilization was available only for these regions.

Demographic and socioeconomic data were obtained from Statistics Sweden’s longitudinal database LISA. Variables of interest for our analysis were age, sex, region of residence, income, education level and (when applicable) year of death. Income was defined as equivalized disposable household income, including income from work and business activities, capital, social benefits and allowances minus final taxes,^21^ and adjusted for the household composition. That is, the household disposable income is related to the size and age of household members. For individuals who died in 2016, data on income and education level of 2015 was used, and to prevent doublecounting we utilized healthcare expenditures only for the remaining months in life in 2016. Children (below age 20) were excluded from the analyses due to lack of data on socioeconomic status. The analyses were approved by the Regional Ethical Review Authority in Gothenburg (#803-17).

### Healthcare utilization and expenditures

Data on visits in primary and specialized outpatient care were obtained from four regional healthcare databases in Västra Götaland, Skåne, Stockholm and Östergötland, including all visits in 2016. Costs for primary and specialized outpatient care visits were calculated based on the average cost for a visit in 2021 in Region Västra Götaland (see Supplementary table A2). We used the costs of 2021 to ensure an up-to-date value of healthcare resources. Relying on costs from one particular region, we aimed to minimize potential bias from cost differences that may be attributed to accounting technicalities.

Inpatient care episodes were received from the Swedish National Board of Health and Welfare’s National Patient Register. Costs related to each care episode in inpatient care were calculated based on prospective diagnosis-related group (DRG) codes. The DRG-cost weight is a relative measure of treatment and the cost of care for an average patient in each DRG group, compiled by the Swedish Municipalities and Regions (SKR).^22^ Using the DRG-weights attained from the patient register, each care episode was multiplied by the base tariff of €6191 in 2021 and summed up to the costs per year for each individual.

Data on purchased prescribed drugs, including date of prescription and total costs, were retrieved from the National Prescribed Drug Register. Prescription drug costs were summed per year for each individual. Due to a lack of data availability, costs for long-term care were omitted from our analysis. All costs were measured in Swedish Krona, converted to Euro (€1 = SEK 10.146, 2021).

### Socioeconomic status

Disposable household income was used to form socioeconomic groups by dividing the income variable into five quintiles (SEG1 to SEG5), stratified by sex and age group. The age groups included ages 20–107 in 5-year age bands, i.e. 20–24 years, 25–29 years, and so on, until individuals of the age of 90 or above entered the last age group. Accordingly, women within a specific age span were grouped in income quintiles by comparison of other women in the same age category, and vice versa for each sex and age group.

### Statistical analysis

Expected lifetime healthcare expenditures were estimated by combining data on survival and average healthcare expenditures (HCE) by age. The analysis was performed using Stata (Version 16.1; StataCorp LLC, 2016).

First, we estimated mean expenditures for each category of healthcare (inpatient, specialized outpatient, primary care and prescription drugs) by sex, income quintile and age, (20–100+, by 1-year bands):


$$\mathrm{average}\_\mathrm{cost}={\bar{\mathrm{HCE}}}_{\mathrm{age},\mathrm{sex},\mathrm{inc}}$$

Fractional polynomial functions were used to descriptively estimate the trends in mean healthcare expenditures, by each category of care, sex and income quintile, with no additional covariates (Stata ‘twoway fpfit’). Compared to regular linear regressions, fractional polynomials better fit the observed data as their process allows for a much more comprehensive range of shapes.^23–25^ In addition, we tested the differences between income quintiles in a fractional polynomial regression (Stata ‘fpreg’).

Next, we estimated mortality rates by age, sex and income quintile. This resulted in income quintile and sex-specific mortality rates, which in turn was used to estimate survival rates and to draw survival curves for each group:


mortality_rateage,sex,inc=∑deathsage,sex,inc∑populationage, sex, inc survivalage,sex,inc=1, age=20 survivalage-1,sex,inc*1-mortalityrateage-1,sex,incage>20 

Weighting the age-mean expenditures by the age-specific survival rates, expected expenditures at each age was estimated by sex and income quintiles. For example, for 64-year-old men in the first (second) income quintile, mean healthcare expenditure was approximately €5123 (€3421), with a survival rate of 0.80 (0.92). Hence, the expected expenditures for a 64-year-old man in the first (second) income quintile was around €4107 (€3142). Finally, expected expenditures were summed across all ages and presented as expected lifetime expenditures, by sex and income quintile. We repeated this estimation separately for each category of care, and for the sum of all categories of care:


$$\begin{array}{c} \mathrm{expecte}{d\_\mathrm{cost}}_{\mathrm{age},\mathrm{sex},\mathrm{inc}}=\mathrm{surviva}l_{\mathrm{age},\mathrm{sex},\mathrm{inc}}\\ \begin{array}{c} \;*\mathrm{averag}{e\_\mathrm{cost}}_{\mathrm{age},\mathrm{sex},inc}\\ \mathrm{expecte}{d\_\mathrm{lifetime}\_\mathrm{cost}}_{\mathrm{sex},\mathrm{inc}}=\sum \mathrm{expecte}{d\_\mathrm{cost}}_{\mathrm{age},\mathrm{sex},\mathrm{inc}} \end{array} \end{array}$$

## Results

### Study population

In total, 440 659 individuals aged 20–107 years [mean age 50.6 years (SD 18.6)] with complete data on income, healthcare utilization and expenditures, year of death and covariates (age/sex) in 2016 were included in the analyses (Supplementary table A3). Women (50.76%) and men (49.24%) had a mean age of 51.49 years and 49.71 years, respectively. The proportion of participants who had a post-secondary education was higher for women than men. The mean disposable household income was higher for men than women across all income quintiles, except for the lowest group (SEG1).

### Trends in mean healthcare expenditures over age

**Figure 1:**
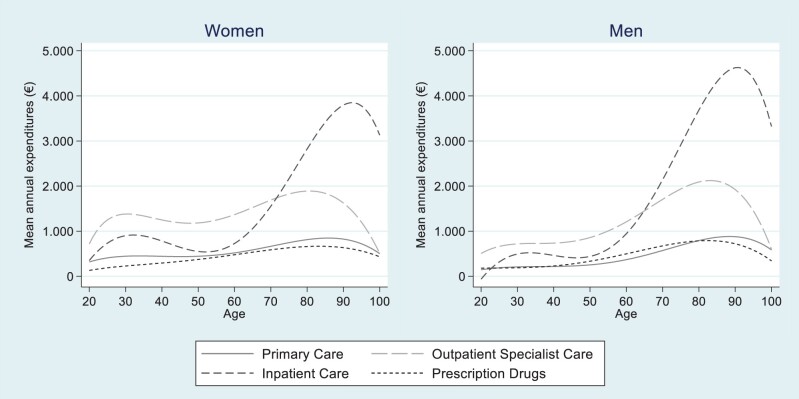
Mean healthcare expenditures (€) over age, by care category and sex

Figure 1 presents the trends in mean expenditures (€) over age, by care category and sex (detailed in Supplementary table A4). From early to the later years of life, the mean expenditures increased in all care categories; however, the increase was most evident for inpatient care between 60 and 90 years. Specialized outpatient care represented the largest expenditure component up to the age of 65 (men) or 70 (women), when there was a shift to higher expenditures of inpatient care. Women demonstrated higher expenditures compared to men between the ages of 20 and 55 years, across all care categories, which is likely related to women’s years of fertility. After age 70, men had higher healthcare expenditures than women for inpatient and specialized outpatient care.

**Figure 2:**
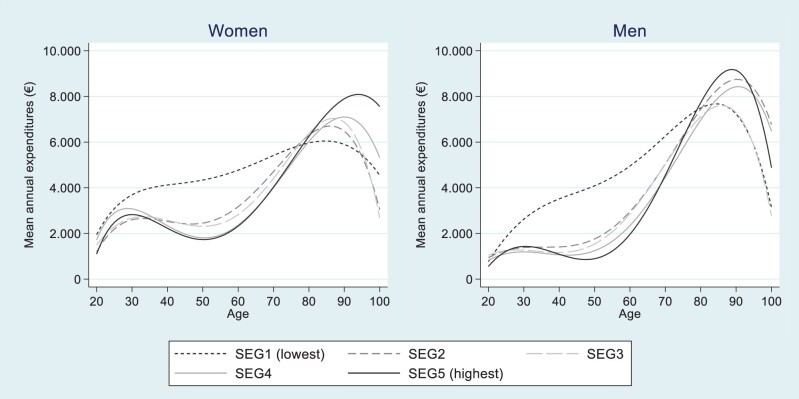
Mean healthcare expenditures (€) over age, by income quintiles and sex

Figure 2 shows the trends over age in total healthcare expenditures (the sum of all categories of care), by sex and income quintile (detailed in Supplementary table A5). Most striking in figure 2 is how the first (lowest) income quintile (SEG1) stands out from the four higher quintiles. Compared with individuals in the fifth (highest) income quintile (SEG5), individuals in the first income quintile indicated twice as high expenditures in ages 45–60. The differences are statistically significant (Supplementary table A6). Figure 2 also displays a dynamic development over age in the association between healthcare expenditures and income. There was a negative association of lower healthcare expenditures the higher the income in ages 40–60, but a positive association in ages above 75 of higher healthcare expenditures the higher the income (corresponding figures in Supplementary table A6). In addition, the healthcare expenditures peaked at around 80 in the first income quintile, approximately five (10) years later for men (women) in the fifth income quintile.

### Estimates of lifetime healthcare expenditures

**Figure 3:**
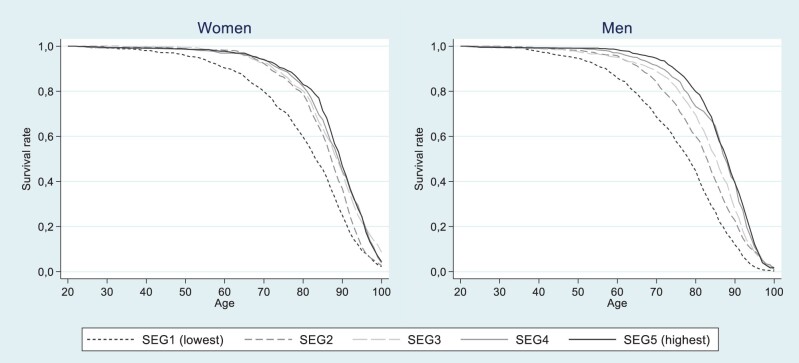
Survival curves over age, by income quintile and sex

Figure 3 displays survival curves by income quintile, for women and men. Unsurprisingly, individuals belonging to the higher income quintiles were expected to live longer than those belonging to the lower quintiles. Similar to figure 2, the first income quintile stands out compared to the four higher quintiles, especially true for women. Across all income levels, women were expected to live longer than men.

**Figure 4:**
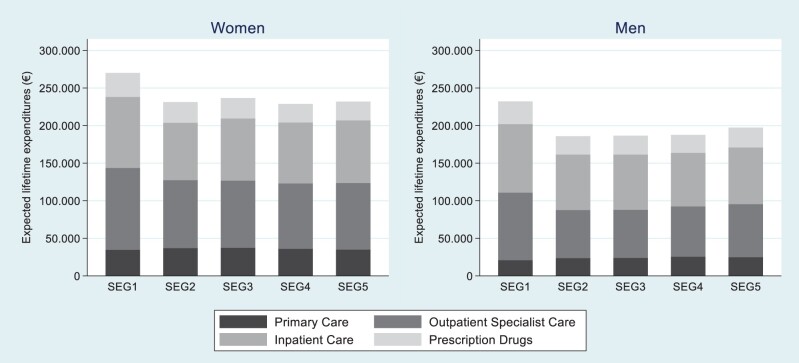
Lifetime healthcare expenditures by care category and income quintile, using income- and sex-specific mortality rates

Combining mean expenditures with the income- and sex-specific survival curves, expected lifetime expenditures by care level, sex and income quintile are illustrated in figure 4, and detailed in Supplementary table A7. Taking income- and sex-specific survival differences into account, individuals in the first income quintile had the highest expected lifetime expenditures compared with the four higher quintiles, true both for men and women. Expected lifetime expenditures in the first income quintile were 17.8% (16.8%) higher than in the fifth income quintile, for women (men). Assuming the same overall survival rate by sex (ignoring socioeconomic differences), expected lifetime expenditures were 38.4% (61.6%) higher for women (men) in the first income quintile compared to the fifth quintile (Supplementary figure A1).

Across all income levels, women had higher expected lifetime expenditures than men. Specifically, the expected lifetime expenditures in the first income quintile were around €270 000 for women (€231 000 for men), and in the fifth income quintile €231 000 for women (€198 000 for men). This is likely the result of women’s improved survival and higher expenditures during reproductive years. Across all income quintiles, specialized outpatient and inpatient care incurred the major components of expected lifetime expenditures. The first income quintile of women had about 30% higher expenditures for outpatient specialized care and about 16% higher expenditures for inpatient care, compared to the fifth income quintile of women. A similar pattern was shown among men. Individuals in the first income quintile had lower expenditures on primary care during a lifetime compared with the other quintiles, although this difference seems to be small.

In robustness analyses, we assessed the impact of our socioeconomic measure on lifetime spending using educational level as the socioeconomic grouping variable. Educational level was divided into three groups: low (lower-secondary), middle (upper-secondary) and high (university) education. We derive a similar spending pattern where individuals with the lowest educational level incurred the highest expenditures over a lifetime; even after accounting for education and sex-specific survival differences (Supplementary figures A2 and A3). Additionally, we performed the analyses based on income deciles (Supplementary figures A4 and A5). Once again, we observed a consistent trend, with the first (lowest) decile incurring the highest lifetime expenditures. We conducted additional analyses using retrospective DRG-weights from the year 2021, which yield marginally different results (Supplementary figures A6 and A7).

## Discussion

### Key findings

This cross-sectional study aimed to describe how lifetime healthcare expenditures are distributed across age, sex and socioeconomic groups, using a sample of approximately 440 000 individuals living in the four largest regions in Sweden. The most prominent finding was the pronounced difference in lifetime healthcare expenditures between individuals at the lowest level (first quintile) of the socioeconomic distribution compared to those with higher socioeconomic status. Accounting for income- and sex-specific survival rates, we find that lifetime expenditures were highest in the first income quintile, despite their evident lower survival rates compared with the other quintiles. In the second to fifth income quintile, the average expected lifetime expenditures were around €230 000 (€187 000) for women (men). In the first income quintile, the expected lifetime expenditures were about 17.9% (16.8%) higher for women (men), specifically, €37 800 (€41 600) higher for women (men).

### Comparison to other studies

Our results correspond to previous findings demonstrating increased use of healthcare resources among groups with lower socioeconomic status.^6^^,^^7^^,^^19^^,^^26–28^ Specifically, our results reflect those of Asaria et al.,^7^ who find a clear socioeconomic gradient in lifetime inpatient expenditures in England but do, however, contradict the results of Kallestrup-Lamb and Marin.^2^ The latter study concludes that differences in lifetime healthcare expenditure across socioeconomic groups in Denmark are small and insignificant after taking mortality differences into account; however, different from our study, their data expands the analysis to include costs for long-term care.

Finding that the lowest income quintile spends the least on primary care over a lifetime is consistent with Kallestrup-Lamb and Marin,^2^ who show that higher socioeconomic groups particularly spend more on primary care physicians over a lifetime than lower socioeconomic groups in Denmark. Prior studies in Sweden have demonstrated differences in the utilization of healthcare services, specifically indicating that groups of lower socioeconomic status, to a larger degree, abstain from seeking healthcare despite perceived medical need.^26^ Consequently, delayed diagnosis and treatment could partly contribute to why some disease-specific mortality is higher among lower socioeconomic groups compared to those of higher status, as diseases might aggravate before receiving medical attention.^26^

Consistent with previous findings from other healthcare settings, we found that in ages 20–55 women had higher expenditures than men, while men generally had higher expenditures than women in the older ages, specifically related to hospital inpatient care.^7–9^^,^^20^ Like most papers that compare estimations of lifetime healthcare expenditures, our results show women spend more than men on each category of care over a lifetime as they tend to live longer lives.^2^^,^^8–10^ Moreover, we observe a negative association of lower healthcare expenditures the higher the income in ages 40–60, but a positive association in ages above 75 of higher healthcare expenditures the higher the income, consistent with Kallestrup-Lamb and Marin.^2^

It is worth noting the pronounced difference in survival rates of the lowest income quintile compared to the rest of the distribution. These results are consistent with previous findings from Sweden, showing the negative relationship between mortality and disposable income to be non-linear, i.e. manifold higher mortality rates in the lowest income quintiles compared to the top half of the distribution.^13^ Similar trends have been shown by educational level.^16^^,^^29^

### Limitations

To our knowledge, this is the first cross-sectional study based on individual-level register data in Sweden to describe how lifetime healthcare expenditures are distributed across socioeconomic groups. We explore expenditures for each category of healthcare based on data from rich national healthcare registers, which minimizes the risk of bias due to incorrect reporting. Our analysis includes expenditures of specialized outpatient, inpatient and primary care, as well as prescription drugs; however, expenses related to long-term care were omitted in the analysis due to data restrictions. Long-term care is a substantial cost component of Swedish health spending.^30^ Considering previous findings from Denmark,^2^ including expenditures of long-term care in our analysis could potentially increase total lifetime expenditures for the higher socioeconomic groups.

A limitation of the current study is that its cross-sectional design assumes that socioeconomic status is static over lifetime. However, as individuals do not necessarily stay within the same income category throughout their lifetime, this may lead to under- or overestimated results.^7^ Further, our analyses did not consider healthcare expenses associated with the period between birth and 20 years of age. For future studies, combining a longitudinal study design with a life cycle perspective may complement our findings.^12^

### Policy implications

When assessing the cost-effectiveness of new interventions, healthcare expenditures are an essential aspect that should be based on an analytical timeframe long enough to capture all effects and expenses related to the intervention, often requiring a lifetime perspective. However, as it is, in practice, rarely feasible to observe expenditures of a study population for the remaining lifetime, estimations of future costs are often based on projections of relatively short periods observed.^8^^,^^9^ The lifetime healthcare expenditure estimates from this study can be used to inform comparative economic models seeking to assess the cost-effectiveness of specific interventions. Furthermore, our analysis shows the distribution of mean healthcare expenditure across age groups. It is clear that average annual costs increase with age and vary between women and men, which should be considered for future assessments of the aging population’s impact on healthcare expenditures.

For many years, research on health inequalities has emphasized the increased risks of disease and premature death in groups of lower income and education,^12^^,^^14^ including in Sweden,^13^^,^^15^^,^^29^ where income, education and work are key factors contributing to these differences.^14^^,^^29^ Briefly looking at the demographics of the different income quintiles in our study sample, we confirm that several key areas of life that have been shown to be important for health differed between the groups. For instance, among men aged 51–55 in the lowest income quintile, about one-third were foreign-born, 50% were unemployed and only 21% had a university degree. Among men in the same age category in the highest income group, one-tenth were foreign-born, 2% were unemployed and 58% had a university degree.

Seeing that lifetime healthcare expenditures of the lowest socioeconomic groups are substantially higher despite their lower life expectancy only acknowledges the inequalities in health that face the most deprived socioeconomic groups. Comparing the findings with those of other studies confirms the association of socioeconomic inequalities in health and increased use of healthcare resources among lower socioeconomic groups.^6^^,^^7^^,^^14^^,^^19^ For policymakers, reducing health disparities and healthcare spending would likely require early efforts to target individuals at the lower level of the socioeconomic distribution. This is especially important, seeing that many of the variations in health at younger ages are associated with socioeconomic status,^12^ and that these groups’ earlier onset of chronic diseases seems to shift rising healthcare expenditures to the younger generations.^7^

The minor differences in lifetime expenditures across the top-four income quintiles found in our study suggest that health improvements targeting the very lowest socioeconomic group may contribute to reduced healthcare expenditures. Improving the health of the most deprived will probably result in enhanced life expectancy. However, since improvements in health most likely would lower healthcare expenditures from a lifetime perspective, we can expect increased life expectancy and better health without necessarily rising healthcare expenditures.^2^

The main finding in this study was that the lowest socioeconomic group had substantially higher healthcare expenditures over a lifetime, despite their shorter life expectancy. Another finding to emerge is that we do not observe a clear social gradient across all income quintiles, but rather markedly higher expenditures in the lowest income quintile compared with the four higher quintiles. As expected, we also found that health care expenditures increase with age, although the pattern between age and spending varied between sexes as well as across socioeconomic groups. The maximum healthcare costs peaked at a higher age for individuals in the relatively higher socioeconomic groups.

## Supplementary Material

ckad140_Supplementary_Data

## Data Availability

The administrative data used in this study are proprietary and we would be violating agreements with government authorities if we distributed the data. The data may be accessed from Swedish national and regional government authorities for researchers that (i) can show that they have ethical approval from an external body for the analyses, and (ii) submit their research plan together with a formal application to the authorities. We are happy to provide detailed information about the process of obtaining the data as well as the full code, going from merging the data sets to analyses. Key points Estimated lifetime healthcare expenditures adjusting for group differences in life expectancy No pure social gradient across income groups, only the lowest quintile emerges Despite lower life expectancy, the lowest income quintile had the highest lifetime expenditures
