# Supplementary material for: Lifetime healthcare expenditures across socioeconomic groups in Sweden
Source: Eur J Public Health. 2023 Aug 30;33(6):994–1000. doi: 10.1093/eurpub/ckad140 (PMC10710355; doi:10.1093/eurpub/ckad140)

Lifetime healthcare expenditures across socioeconomic groups in Sweden

Appendix

Table A1 Descriptive statistics of Swedish regions 1
Table A2 Average cost per visit to physician or other health personnel in primary and specialized outpatient care in 2021. 2
Table A3 Characteristics of the study population. 3
Table A4 Mean annual expenditures (€) by care level, age groups, and sex 4
Table A5 Mean annual total healthcare expenditures (€) by socioeconomic group, age group, and sex. 5
Table A6 Estimated difference between income quintiles, in annual healthcare expenditures (€) 6
Table A7 Lifetime healthcare expenditures (€) by care category, income quintile, and sex, using income- and sex-specific mortality rates. 7
Figure A1 Overall mortality rate - Lifetime healthcare expenditures (€) by care level and socioeconomic group 8
Figure A2 Education level - Lifetime healthcare expenditures by care level, sex, and education level 9
Figure A3 Education level - Mean annual healthcare expenditures over age, by sex and educational level 10
Figure A4 Income deciles - Mean annual healthcare expenditures over age, by sex and income group 11
Figure A5 Income deciles - Lifetime healthcare expenditures by care level, sex, and income group 12
Figure A6 Retrospective DRG-weights - Mean annual expenditures (€) over age, by care category and sex 13
Figure A7 Retrospective DRG-weights – Lifetime healthcare expenditures by care level, sex, and income group 14

Table A1 Descriptive statistics of Swedish regions

				Education (%)					
	Inhabitants	Women (%)	Age, median	Lower second.	Upper second.	Post-second.	Per capita income (€)	Per capita HCE (€)	Care need index
Sweden	9 995 153	49.8	41.2	12.0	44.0	42.0	24 366	2 665	1.00
Region Skåne	1 324 565	50.2	40.9	12.5	42.1	43.0	23 416	2 585	1.08
Region Stockholm	2 269 060	50.1	39.1	10.6	35.8	50.7	27 347	2 766	1.06
Region Östergötland	452 105	49.5	41.3	12.5	45.8	40.2	23 448	2 610	1.03
Region Västra Götaland	1 671 783	49.8	41.0	12.4	43.7	42.2	24 452	2 502	1.01
Region Blekinge	158 453	49.0	43.1	12.2	48.1	38.1	22 444	2 914	1.01
Region Dalarna	284 531	49.5	43.5	13.5	52.6	32.5	22 903	2 717	0.98
Region Gotland	58 003	50.2	44.6	12.9	52.0	34.0	21 749	3 024	0.91
Region Gävleborg	284 586	49.6	43.4	14.4	52.3	32.2	22 497	2 840	1.12
Region Halland	320 333	49.9	42.1	11.8	47.6	39.3	25 093	2 621	0.88
Region Jämtland Härjedalen	128 673	49.5	43.2	10.4	52.2	36.1	22 561	2 907	0.98
Region Jönköpings county	352 735	49.6	41.5	14.5	49.3	34.8	24 121	2 616	0.96
Region Kalmar	242 301	49.5	43.8	13.9	50.4	34.0	22 721	2 741	1.00
Region Kronoberg	194 628	49.2	41.6	13.1	47.2	37.6	23 565	2 672	1.01
Region Norrbotten	250 570	48.9	43.7	9.4	53.7	35.6	23 629	3 013	0.90
Region Sörmland	288 097	49.9	42.3	15.1	50.2	33.1	23 202	2 706	1.16
Region Uppsala	361 373	50.1	40.2	10.4	41.1	46.7	24 740	2 508	0.98
Region Värmland	279 334	49.8	43.5	11.4	51.5	35.0	22 882	2 740	0.98
Region Västerbotten	265 881	49.5	41.7	8.5	46.7	43.6	23 213	2 781	0.89
Region Västernorrland	245 572	49.6	43.5	11.7	51.8	35.3	23 149	2 872	1.00
Region Västmanland	267 629	49.7	42.2	13.4	48.8	36.4	23 790	2 810	1.12
Region Örebro county	294 941	49.9	41.7	13.2	49.4	36.1	23 074	2 738	1.08

Note. Statistics collected from the Swedish municipality and region database Kolada (www.kolada.se). Per capita income refers to annual median income in year 2016, converted to Euros (Price year 2021, 1 € = 10,146 SEK). Per capita HCE refers to healthcare expenditures in year 2016, defined as the total net costs of healthcare, including dental care and prescribed drugs, divided by the number of inhabitants, converted to Euros. Care need index is an index constructed by socioeconomic parameters to defined an area’s risk for ill-health, a value of 1.00 implies higher risk for ill-health compared to the nation and vice versa.

Table A2 Average cost per visit to physician or other health personnel in primary and specialized outpatient care in 2021.

Care level	Average cost per visit (€)
**Primary care**	
Visit physician	171
Visit other health personnel	70
**Specialized outpatient care**	
Visit physician	564
Visit other health personnel	277

Note. Price year 2021, 1 € = 10,146 SEK (Swedish Krona).

Table A3 Characteristics of the study population.

	Women	Men	Total study population
Population size (*n*)	223,661	216,998	440,659
%	50.76 %	49.24 %	100.00 %
20-24	7.66 %	8.17 %	7.91 %
25-29	8.59 %	9.17 %	8.88 %
30-34	7.39 %	7.96 %	7.67 %
35-39	7.59 %	7.94 %	7.77 %
40-44	8.26 %	8.76 %	8.50 %
45-49	8.48 %	8.91 %	8.69 %
50-54	8.81 %	9.28 %	9.04 %
55-59	7.61 %	8.07 %	7.84 %
60-64	7.28 %	7.45 %	7.37 %
65-69	7.56 %	7.36 %	7.46 %
70-74	7.16 %	6.91 %	7.04 %
75-79	4.94 %	4.37 %	4.65 %
80-84	3.64 %	2.88 %	3.27 %
85-89	2.81 %	1.81 %	2.32 %
90+	2.21 %	0.97 %	1.60%
Age in years, mean and SD	51.49 (19.1)	49.71 (18.1)	50.62 (18.6)
Highest obtained educational level (%)			
Lower-secondary	15.97 %	17.70 %	16.82 %
Upper-secondary	41.18 %	45.78 %	43.45 %
University	42.84 %	36.52 %	39.73 %
Household income (€) mean and SD by SES group			
SEG1	15 232 (9 293)	14 220 (27 492)	14 734 (20 400)
SEG2	27 324 (9 266)	29 263 (6 971)	28 279 (8 272)
SEG3	39 396 (13 894)	43 136 (10 749)	41 389 (12 563)
SEG4	54 592 (17 225)	58 626 (14 161)	56 579 (15 919)
SEG5	114 990 (372 045)	118 589 (216 729)	116 762 (305 599)

Note. Education level is operationalized as the highest obtained educational degree starting with lower-secondary education (compulsory nine years or less) upper-secondary education (three years or less), university (two years or higher). Price year 2021, 1 € = 10.146 SEK.

Table A4 Mean annual expenditures (€) by care level, age groups, and sex.

Age groups	Primary care	Outpatient specialist care	Inpatient care	Prescription drugs
**Women**				
20-24	362	981	542	160
25-29	405	1264	772	197
30-34	469	1442	1016	221
35-39	452	1313	750	303
40-44	427	1170	576	307
45-49	434	1199	568	337
50-54	470	1240	614	393
55-59	501	1279	738	438
60-64	537	1338	975	481
65-69	577	1518	1281	565
70-74	679	1802	1725	591
75-79	803	1973	2227	677
80-84	861	1970	2946	673
85-89	857	1731	3682	645
90+	717	1254	3888	582
**Men**				
20-24	179	590	305	170
25-29	184	650	278	148
30-34	196	680	334	250
35-39	209	803	340	210
40-44	224	741	423	232
45-49	246	792	471	276
50-54	283	923	634	356
55-59	346	1106	1011	475
60-64	405	1235	1238	531
65-69	468	1485	1746	622
70-74	590	1823	2248	710
75-79	732	2109	2873	734
80-84	831	2075	3672	802
85-89	923	2138	4558	793
90+	801	1587	4795	636

Note. Price year 2021, 1 € = 10,146 SEK. Individuals equal to or above the age of 90 are categorized in the same age group.

Table A5 Mean annual total healthcare expenditures (€) by socioeconomic group, age group, and sex.

Age groups	SEG1	SEG2	SEG3	SEG4	SEG5
**Women**					
20-24	2525	1879	2115	2258	1614
25-29	3407	2080	2231	3524	2821
30-34	4075	2533	3346	2976	3030
35-39	3903	2774	2515	2132	2114
40-44	4165	2468	2144	1761	1789
45-49	4273	2568	2309	1962	1767
50-54	4438	2476	2605	2123	1969
55-59	4729	2894	2826	2491	2094
60-64	4821	3470	3287	3018	2831
65-69	5392	3944	3828	3512	3523
70-74	5764	5607	4654	4570	4546
75-79	5623	6430	5827	5358	5938
80-84	6169	6849	6861	6221	6408
85-89	6046	6643	7338	7149	7813
90+	5406	5532	5928	7374	7969
**Men**					
20–25	1451	1065	1376	1172	1158
26–30	1985	1142	909	1048	1217
31–35	2707	1424	1133	959	1076
36–40	3389	1254	1217	962	986
41–45	3466	1461	1194	1041	933
46–50	3488	1728	1414	1249	1043
51–55	4526	1924	1754	1510	1267
56–60	5547	2602	2656	2013	1866
61–65	5140	3349	3335	2799	2423
66–70	5976	4164	4095	3767	3602
71–75	5816	5695	5605	4838	4899
76–80	6765	6388	6286	6314	6486
81–85	7830	7875	7042	6525	7631
85–89	7605	8587	7913	8453	9504
90+	6509	8721	6747	8291	8833

Note. Price year 2021, 1 € = 10,146 SEK.

Table A6 Estimated difference between income quintiles, in annual healthcare expenditures (€)

	**Women**	**Men**
	Coeff. (95% CI)	Coeff. (95% CI)
SEG1	775.35***	1001.81***
	(421.87; 1128.83)	(546.37; 1457.25)
SEG2	–149.98	181.98
	(–503.48; 203.52)	(–275.47; 639.43)
SEG3	–84.05	–198.04
	(–437.53; 269.42)	(–655.54; 259.46)
SEG4	–135.99	–65.91
	(–491.55; 219.57)	(–523.40; 391.59)
N	432	416

	**Women <80 years**	**Women ≥80 years**	**Men <80 years**	**Men ≥80 years**
	Coeff. (95% CI)	Coeff. (95% CI)	Coeff. (95% CI)	Coeff. (95% CI)
SEG1	1583.83***	–1060.43*	1943.56***	–1483.21*
	(1395.79; 1771.86)	(–2078.89; –41.97)	(1740.47; 2146.65)	(–2966.25; –0.17)
SEG2	403.79***	–1418.89**	450.41***	–575.21
	(215.75; 591.82)	(–2438.52; –399.27)	(247.31; 653.50)	(–2069.95; 919.54)
SEG3	265.72**	–900.5	335.30**	–1682.22*
	(77.68; 453.75)	(–1918.96; 117.97)	(132.21; 538.39)	(–3179.71; –184.73)
SEG4	102.56	–665.09	67.95	–507.95
	(–85.47; 290.59)	(–1704.19; 374.02)	(–135.15; 271.04)	(–2005.45; 989.54)
N	300	132	300	116

Note. Estimated by fractional polynomial regression with a fourth-degree fractional polynomial of age, regressing the expected cost on age and income quintile. SEG – socioeconomic group defined by income quintiles. The fifth (highest) income quintile was used as the reference. Price year 2021, 1 € = 10,146 SEK. * $p < 0.05$, ** $p < 0.01$, *** $p < 0.001$

Description Table A6: The (average) difference in annual healthcare expenditures, compared to SEG5. Women in the lowest income quintile had on average €775 (95% CI 422; 1129) higher expenditures compared to the highest income quintile. The equivalent figure for men was €1002 (95% CI 546; 1457).

Stratified by age, women below 80 years in the lowest income quintile, has statistically significant higher annual healthcare expenditure compared to the highest income quintile. While for women 80 years and above, healthcare expenditures are statistically significantly lower in the lowest income quintile compared to the highest income quintile.

Table A7 Lifetime healthcare expenditures (€) by care category, income quintile, and sex, using income- and sex-specific mortality rates.

Care level	SEG1	SEG2	SEG3	SEG4	SEG5
**Women**					
Primary care	34 698	36 986	37 363	36 017	34 952
Outpatient specialist care	108 934	90 431	89 326	87 056	88 690
Inpatient care	94 609	76 306	82 821	81 163	83 269
Prescription drugs	31 920	27 601	27 238	24 690	25 057
Total (€)	270 160	231 325	236 749	228 926	231 968
**Men**					
Primary care	20 846	23 628	23 960	25 522	24 872
Outpatient specialist care	89 917	64 081	64 017	66 903	70 716
Inpatient care	91 337	73 746	73 517	71 352	75 379
Prescription drugs	30 113	24 478	25 075	23 828	26 480
Total (€)	232 214	185 934	186 569	187 605	197 447

Note. Price year 2021, 1 € = 10,146 SEK.

Figure A1 Overall mortality rate - Lifetime healthcare expenditures (€) by care level and socioeconomic group

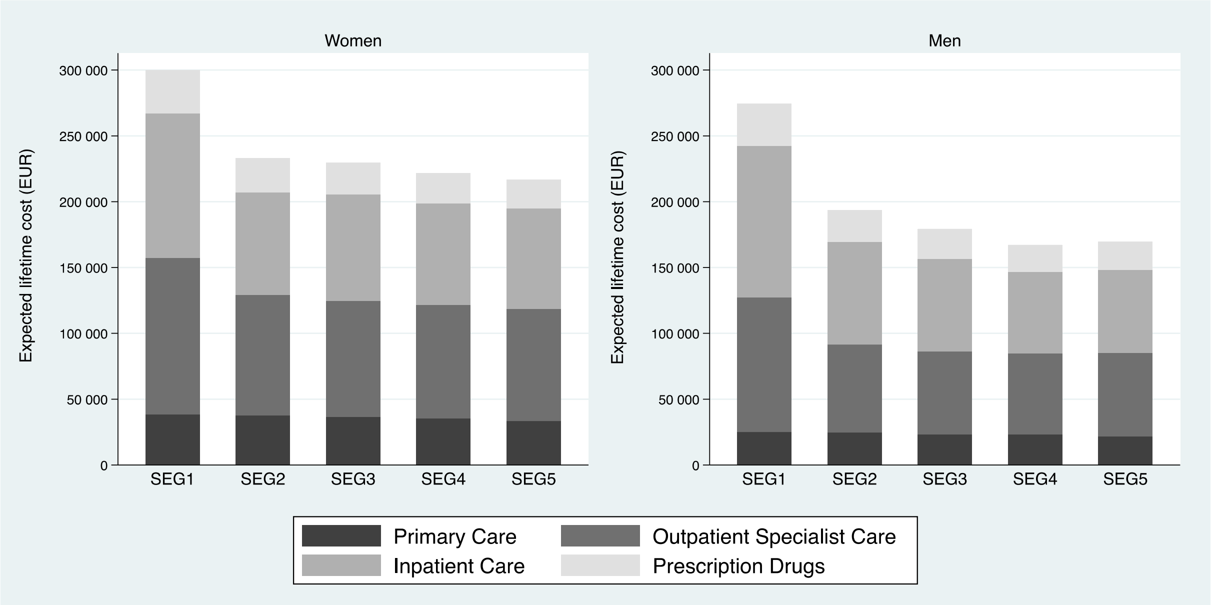

Note. Expected lifetime healthcare expenditures calculated assuming the same overall mortality rate for all women and men

Figure A2 Education level - Lifetime healthcare expenditures by care level, sex, and education level

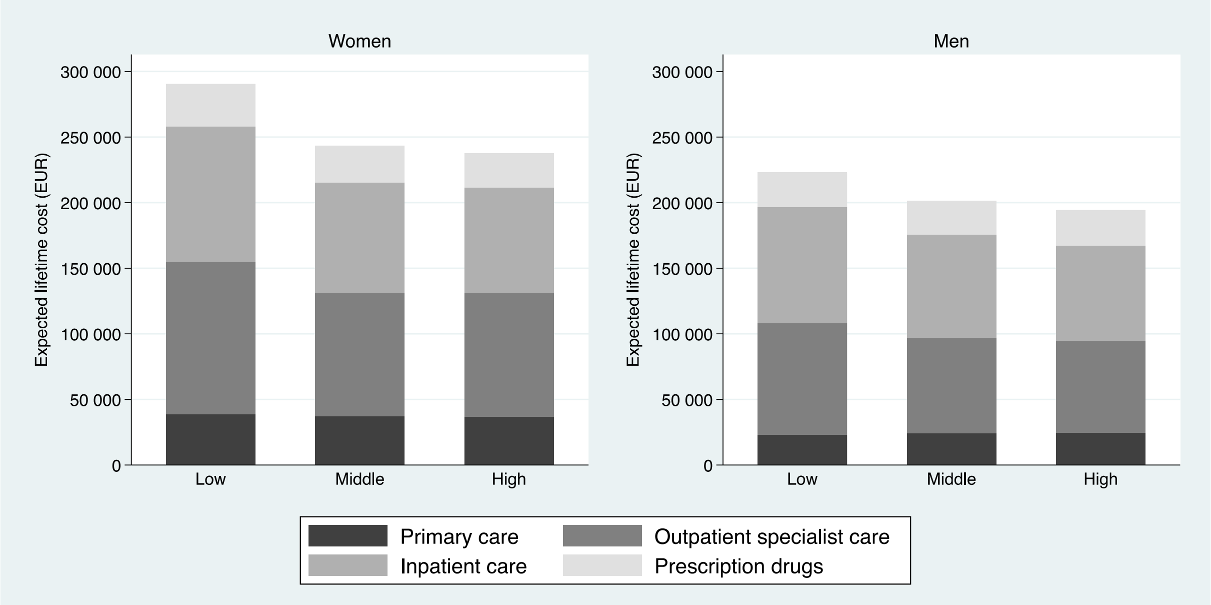

Note. Educational level divided into three groups starting with low (lower secondary), middle (upper secondary), and ending with high (post-secondary).

Figure A3 Education level - Mean annual healthcare expenditures over age, by sex and educational level.

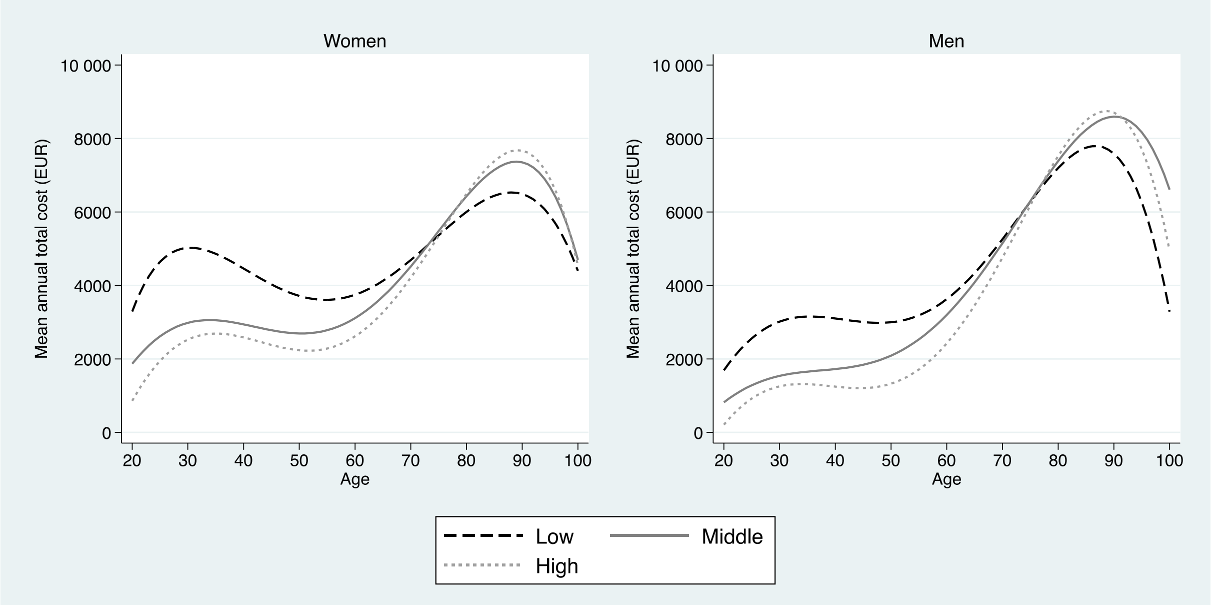

Note. Educational level divided into three groups starting with low (lower secondary), middle (upper secondary), and ending with high (post-secondary).

Figure A4 Income deciles - Mean annual healthcare expenditures over age, by sex and income group

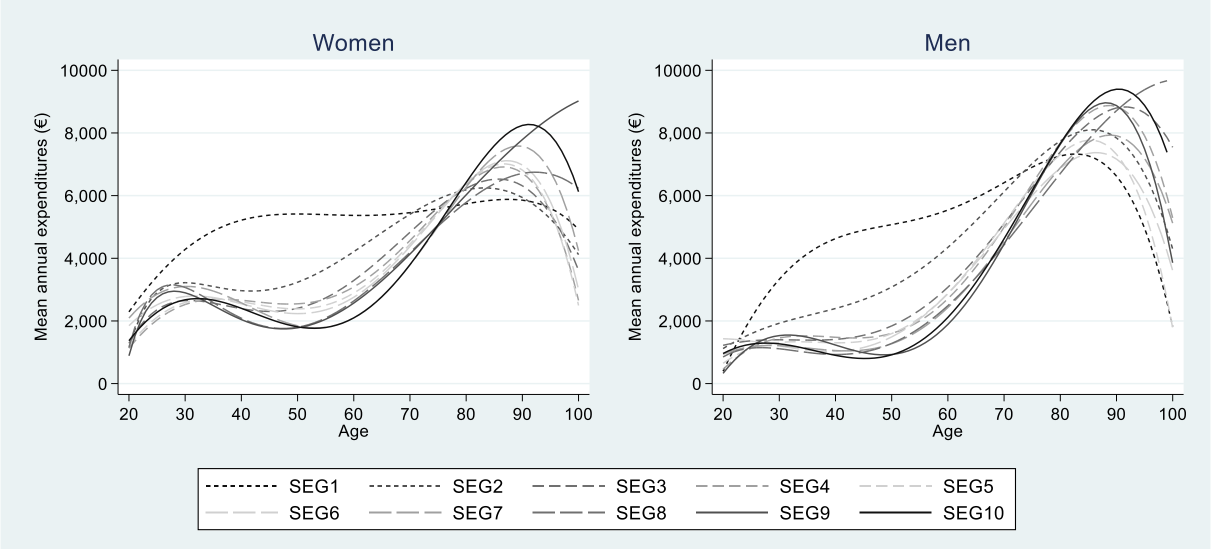

Figure A5 Income deciles - Lifetime healthcare expenditures by care level, sex, and income group

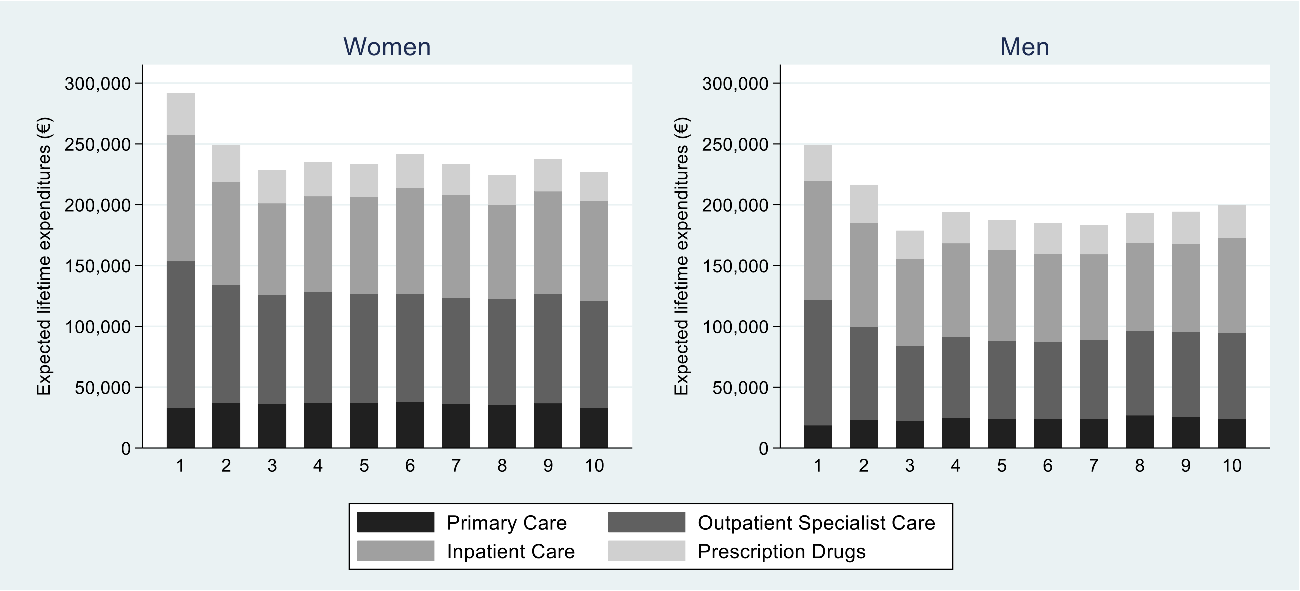

Figure A6 Retrospective DRG-weights - Mean annual expenditures (€) over age, by care category and sex

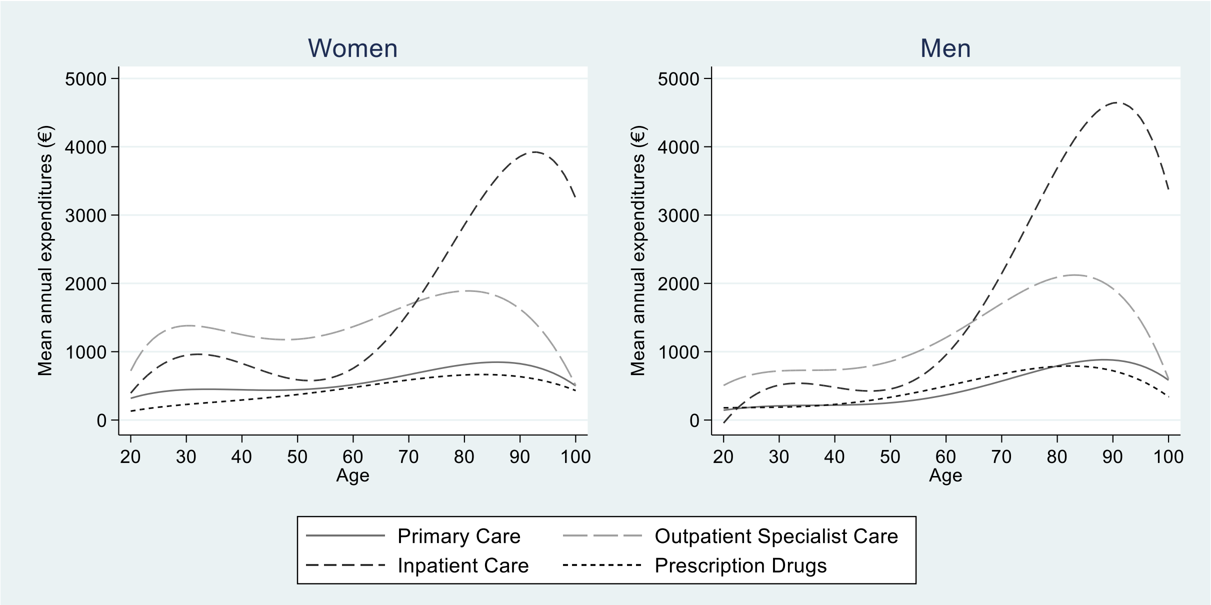

Figure A7 Retrospective DRG-weights – Lifetime healthcare expenditures by care level, sex, and income group

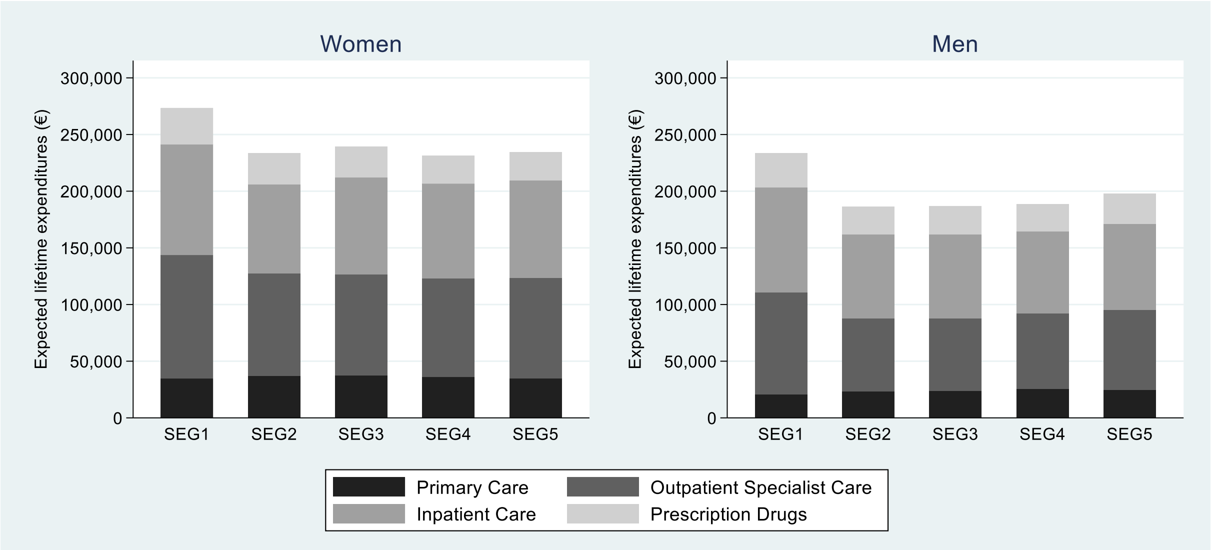

Supplement: ckad140_Supplementary_Data [file ckad140_supplementary_data.pdf]
